# Askin tumor mimicking a hydatid cyst of the lung in children: case report

**DOI:** 10.11604/pamj.2014.17.131.1056

**Published:** 2014-02-25

**Authors:** El Hassane Kabiri, Abderrahmane Al Bouzidi, Meryem Kabiri

**Affiliations:** 1 Mohamed V Military Teaching Hospital, Morocco; 2 Department of Pediatrics Childrens hospital, Faculté de médecine et de Pharmacie, Hay Riad – Rabat 10100, Morocco

**Keywords:** Askin tumor, chemotherapy, surgery

## Abstract

Askin tumor is a malignant small round cell tumor that originates from the thoracopulmonary region, usually observed in young subjects. We report a case of askin tumor in a 11 year-old men, who had in his past history a surgery for hydatid cyst of the liver, actually hospitalised for right pulmonary upper lobe mass of the lung. After resection of the mass, the pathological exam confirmed the diagnosis of primitive neuroectodermal tumor (PNET). An adjuvant treatment (radiotherapy and chemotherapy) was done; the patient died 11 months after surgery. Askin tumor is an exclusion diagnosis not always readily made.

## Introduction

In 1979, Askin and al first identified a PNET of the thoracopulmonary region in children and young adults as a specific pathological entity [1]. Askin tumors account for approximately 6. 5% of all PNETs, and arises in the periosteum, soft tissue, and extrapulmonary fields of the thoracic wall and in the lung. Over the last decade, diagnosis has greatly improved with the introduction of an array immunohistochemical markers. But, this tumor still raises many questions about its individualisation and its link with Ewing's sarcoma (ES). Uniform treatment regimen remains undetermined. We report a case of askin tumour mimicking pulmonary hydatid cyst that is endemic in Morocco.

## Patient and observation

**Figure 1:**
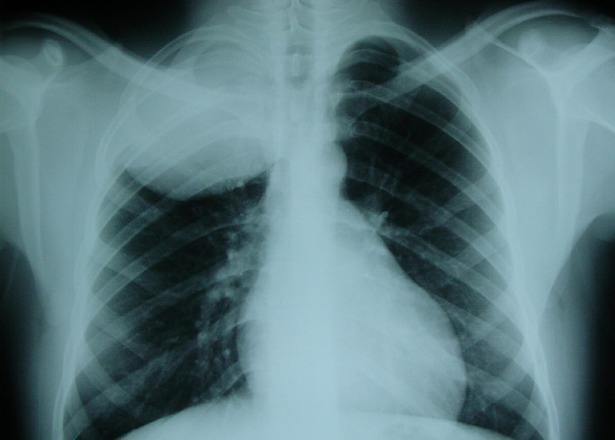
Chest x-ray revealed a voluminous opacity of the right upper pulmonary lobe

**Figure 2:**
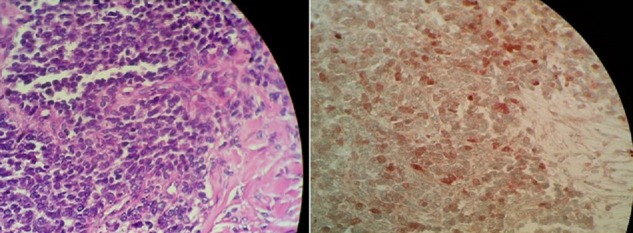
Histological analysis of the mass

A 11 year-old child was referred to our department with a 1 month history of pain in the right chest. In his past history, he had a surgery of hydatid cyst of the liver three years ago. A physical examination revealed a diminution of the breath sound in the upper right lobe without other clinical sign, and the patient was in excellent condition. The chest X-ray realised in our patient showed right upper lobe opacity (Figure 1) and the first diagnosis suspected, within his past history and endemic aspect of hydatid disease in our region was a hydatid cyst of the lung. The patient was operated and we discovered tissular mass invading upper lobe with suspect parietal contact, we performed a complete resection of the mass with dorsal right upper lobe segmentectomy and resection of parietal pleural beside the tumour., The diagnosis of askin's tumor was established histological (Figure 2) on the presence of small cells with scant cytoplasm and focal necrosis, the cells were round and had scant cytoplasm with inconspicuous nucleoli. Mitotic figures were numerous. Rosettes were only focally identified, and immunohistochemistry objective that the tumor cells is positive for neuron-specific enolase (NSE) and CD99. Our patient receives adjuvant chemoradiotherapy with good evolution. Two years after the patient died with lung, liver and bone metastasis.

## Discussion

Askin's tumor or primitive neuroectodermal tumor (PNET), is an extremely malignant small cell tumor of the thoracopulmonary region. Originating from the neuroepithelial cells of the intercostals nerves, these tumors may involve the ribs, the soft tissue of the chest wall, and the lung. An indistinguishable t (11,22) translocation has been demonstrated in Askin's and Ewing's tumour, perhaps indicating a common origin for these tumours [1, 2], and PNET's are members of Ewing sarcomas family tumours (ESFT). Askin tumor may present as a chest wall mass with thoracopulmonary symptoms such as chest pain, cough, or dyspnea. In our patient, who presented a thoracic pain, upper right lung mass was discovered, and because of his past history of surgery for hydatid cyst of the liver, three years ago, the first diagnosis evocated was a lung localisation of the hydatid cyst. The radiological characteristics of askin tumours are non specific, its might associated with inspissations pleural in 70% or effusion in 28% and rib involvement in 40% of cases. Differential diagnosis has to be made with other small cell tumors such as Ewing's sarcoma, rhabdomyosarcoma, malignant lymphoma, and neuroblastoma [3, 4]. Histologically, it contains homogeneous small round-to- spindle cells growing in compact sheets on pseudo-lobules, and exhibit neurosercretory granules and cell. In ultra structures studies, occasionally rosette-like structures were evident but the lack of typical Homer-Wright rosettes excluded neuroblastoma. By immunohistochemistry, the tumor was strongly positive for neuron-specific enolase (NSE), which is a specific marker for neural elements, even though Ewing's sarcoma and rhabdomyosarcoma may display NSE positive. Genetically, detection of EWS/FLI-1 fusion gene transcripts has recently become a useful tool for the diagnosis of PNET/ES [5]. In our case, the diagnostic of askin tumor was confirmed by histology (Figure 2)

Marina et al [6] felt there is a need at least two or three of these criteria for retain the diagnosis of askin's tumors: 1) presence of rosette of homer Wright, 2) origin from a peripheral nerve, 3)the positive of NSE or Leu-7, 4) existence of cytoplasmic extensions, neurosecretory granules and microtubules, 5) the translocation t (11, 22) q (24, 12), 6)detection of proto-oncogenes (N-myc, C-myb, C-ets-1), 7) an activity of biosynthetiques enzymes of the neurotransmitters (tyrosine hydroxylase, dopamine b hydroxylae and acetylcholine transferase).

Multimodality therapy has improved local control and overall survival for patients with PNET of the chest wall, but uniform treatment regimens remains underteminated. Actually, standard treatment of askin's tumor comprises surgery after chemotherapy [7]. The prognosis in Askin tumor is very poor despite the combination of chemotherapy, radiotherapy, and surgical removal; with a 2 year survival of 38% [2]. Some authors suggest that aggressive treatment with preoperative chemotherapy, radical surgical resection, postoperative chemotherapy, and irradiation is the best approach to Askin tumor [7].

## Conclusion

Askin tumors are rare but highly malignant and must treated by aggressive interdisciplinary management including radical surgery and chemotherapy.
